# Ceftobiprole alone versus ampicillin-ceftriaxone against borderline-penicillin-resistant, ampicillin-susceptible, and vancomycin-resistant *Enterococcus faecalis* isolates

**DOI:** 10.1128/aac.01050-25

**Published:** 2025-11-24

**Authors:** Olivia Gladys Funk, Jingyi Li, Ifra Khan, Jaclyn A. Cusumano

**Affiliations:** 1 Division of Pharmacy Practice, Arnold and Marie Schwartz College of Pharmacy and Health Sciences, Long Island University, Brooklyn, New York, USA; The Peter Doherty Institute for Infection and Immunity, Melbourne, Victoria, Australia

**Keywords:** pharmacokinetics, *in vitro*, endocarditis, infective endocarditis, *Enterococcus faecalis*, enterococcus, ceftobiprole, penicillin resistance, vancomycin resistance, vancomycin

## Abstract

Despite the use of recommended treatments against *Enterococcus faecalis* infective endocarditis, mortality rates remain at 30%. Penicillin-resistant, ampicillin-susceptible *E. faecalis* (PRASEF) has been associated with worsened clinical outcomes; however, a more common phenotype, borderline-PRASEF (penicillin minimum inhibitory concentration [MIC] 4–8 µg/mL), decreases the activity of ampicillin-ceftriaxone *in vitro*. Ceftobiprole presents a promising alternative against borderline-PRASEF and also has shown activity against vancomycin-resistant *E. faecalis* (VREfs). The ceftobiprole MIC distribution was determined via broth microdilution for 78 *E. faecalis* clinical blood isolates, including 71 borderline-PRASEF. Ceftobiprole activity alone was compared to ampicillin-ceftriaxone via *in vitro* 24-hour time-kill assays against 30 *E. faecalis* isolates, 24 of which were borderline-PRASEF and 15 were VREfs. Ceftobiprole and ceftriaxone were tested at physiologic concentrations (*fCpss* 13.9 µg/mL and 17.2 mcg/mL, respectively) and ampicillin at subinhibitory concentrations (0.25 × MIC and 0.5 × MIC). Isolates where ceftobiprole alone had limited activity were tested against ceftobiprole-ampicillin. Ceftobiprole maintained activity against borderline-PRASEF compared to ampicillin-ceftriaxone. Ceftobiprole also demonstrated more activity against VREfs isolates and isolates with an elevated ceftobiprole MIC ≥16 µg/mL, compared to ampicillin-ceftriaxone. Ceftobiprole did not achieve ≥2 log_10_ CFU/mL kill in six isolates but was able to achieve this once combined with ampicillin. When tested at ampicillin inhibitory concentrations, ceftobiprole-ampicillin activity trended towards antagonism in two isolates. Overall, ceftobiprole alone had greater activity compared to ampicillin-ceftriaxone, and there may be a potential role of ceftobiprole combination therapy in certain isolates.

## INTRODUCTION

*Enterococcus faecalis* accounts for 90% of enterococcal infective endocarditis (IE) and is the leading causative pathogen in transcatheter aortic valve implant-associated IE (1–3). Due to the severity of *E. faecalis* IE, guidelines recommend treatment with two agents to achieve bactericidal activity (i.e*.,* ampicillin plus ceftriaxone or ampicillin/penicillin plus gentamicin) (1, 4). Gentamicin-based combinations have fallen out of favor due to increasing resistance and the risk of nephrotoxicity (5–9). However, despite a shift to ampicillin-ceftriaxone therapy, mortality rates are consistently up to 30% (1, 10–12).

The emergence of penicillin-resistant, ampicillin-susceptible *E. faecalis* (PRASEF) has further complicated treatment due to an association with increased mortality (13). The mechanism driving this may be due to a mutation in the penicillin-binding protein-4 (*pbp4*) promoter region that results in PBP4 upregulation, which is the primary binding site for ampicillin (13, 14). PRASEF is currently not common in the United States; however, borderline-PRASEF (penicillin minimum inhibitory concentration [MIC] 4–8 µg/mL; Clinical & Laboratory Standards Institute [CLSI] susceptibility breakpoint ≤8 µg/mL [15]) has been described in 25% of blood isolates across a New York City health system (16, 17). This high prevalence of borderline-PRASEF raises concern as ampicillin-ceftriaxone, *in vitro*, has shown decreased synergy and bactericidal activity against borderline-PRASEF isolates (18). PBP4 upregulation was also described in one borderline-PRASEF isolate, which also had higher beta-lactam MICs (19). Although the clinical implications of borderline-PRASEF isolates are unknown, alternative treatments against *E. faecalis* IE should be explored.

Ceftobiprole is a recently FDA-approved extended-spectrum cephalosporin, which, unlike other cephalosporins, has activity against enterococci, including vancomycin-resistant enterococci (VRE) (14, 20). The activity against *E*. *faecalis*, including vancomycin-resistant *E. faecalis* (VREfs), is likely due to high affinity for PBP4, similar to ampicillin (14). The activity of ceftobiprole against borderline-PRASEF isolates has not been evaluated.

Clinically, ceftobiprole has only been evaluated in combination with ampicillin against *E. faecalis* infections and showed high clinical cure and microbiologic eradication rates (21, 22). No data exist on the clinical use of ceftobiprole as monotherapy against *E. faecalis*.

The objective of this study was to evaluate the activity of ceftobiprole alone versus ampicillin-ceftriaxone against *E. faecalis*, including borderline-PRASEF and VREfs isolates, and to determine the activity of ampicillin when added to ceftobiprole.

## RESULTS

### Isolate susceptibilities

**Fig 1:**
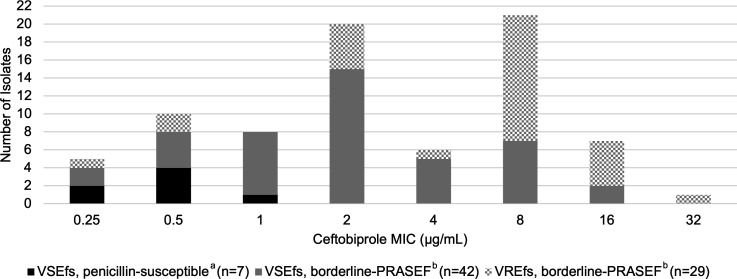
MIC distribution of *E. faecalis* isolates by BMD (*N* = 78). Abbreviations: PRASEF, penicillin-resistant, ampicillin-susceptible *E. faecalis*; VREfs, vancomycin-resistant *E. faecalis*; VSEfs, vancomycin-susceptible *E. faecalis*. ^a^Penicillin-susceptible: MIC ≤ 2 µg/mL. ^b^Borderline-PRASEF: MIC 4–8 µg/mL.

The ceftobiprole MIC distribution by broth microdilution (BMD) of 78 *E. faecalis* isolates is illustrated in Fig. 1. The MIC_50_ and MIC_90_ were 2 and 16 µg/mL, respectively. VREfs isolates had higher ceftobiprole MICs compared to vancomycin-susceptible *Enterococcus faecalis* (VSEfs) isolates with MIC_50_ of 8 versus 2 µg/mL and MIC_90_ of 16 versus 8 µg/mL, respectively. The individual ampicillin, penicillin, ceftriaxone, ceftobiprole, and vancomycin MICs of the 30 isolates included in the time-kill analysis are outlined in Table S1.

### Time-kill analysis

**Fig 2:**
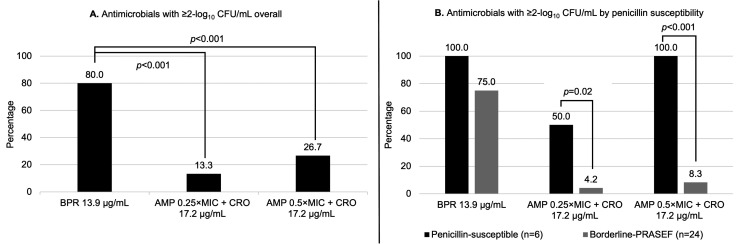
Ceftobiprole alone versus ampicillin-ceftriaxone combinations which achieved ≥2-log_10_ CFU/mL against *E. faecalis* blood isolates via 24-hour time-kill assays (*N* = 30); (**A**) comparison of ceftobiprole vs ampicillin-ceftriaxone in all isolates; (**B**) comparison of ceftobiprole vs ampicillin-ceftriaxone by penicillin susceptibility. Abbreviations: AMP, ampicillin; BPR, ceftobiprole; CRO, ceftriaxone; PRASEF, penicillin-resistant, ampicillin-susceptible *E. faecalis*. ^a^Ceftobiprole was tested at the free plasma steady-state concentration (*f*Cp_SS_ = 13.9 µg/mL) based on population pharmacokinetic data for a 500 mg IV q8h regimen. ^b^Ceftriaxone was tested at the free plasma steady-state concentration (*f*Cp_SS_ = 17.2 µg/mL) based on population pharmacokinetic data for a 2 g IV q12h regimen. ^c^Penicillin-susceptible: MIC ≤ 2 µg/mL. ^d^Borderline-PRASEF: MIC 4–8 µg/mL.

Among 30 *E. faecalis* clinical blood isolates, ampicillin or ceftriaxone alone did not have antimicrobial activity against any isolates. Ceftobiprole alone achieved ≥2-log_10_ CFU/mL kill more often than ampicillin 0.25 × MIC and 0.5 × MIC plus ceftriaxone combinations (80.0% vs. 13.3% [*P* < 0.001] vs. 26.7% [*P* < 0.001], respectively) (Fig. 2A). Ceftobiprole alone also demonstrated greater median log_10_ CFU/mL change from starting inoculum compared to ampicillin 0.25 × MIC and 0.5 × MIC plus ceftriaxone combinations (−3.4 [interquartile range, IQR: −3.9 to −2.7] vs. 0.7 [IQR, 0.2–1.4; *P* < 0.001] vs. −0.8 [IQR, −2.0 to 0.2; *P* < 0.001], respectively).

When compared by penicillin susceptibilities, ceftobiprole alone maintained similar ≥2-log_10_ CFU/mL kill against both penicillin-susceptible vs. borderline-PRASEF isolates (100% vs. 75.0%, *P* = 0.30, Fig. 2B). While ceftobiprole alone demonstrated greater median log_10_ CFU/mL change in penicillin-susceptible isolates compared to borderline-PRASEF isolates (−4.2 [IQR, −4.2 to −4.0] vs. −3.0 [IQR, −3.7 to −2.4; *P* = 0.001], respectively). Ampicillin 0.25 × MIC plus ceftriaxone demonstrated significantly greater attainment of ≥2-log_10_ CFU/mL kill and median log_10_ CFU/mL change against penicillin-susceptible vs. borderline-PRASEF isolates (50.0% vs. 4.2% [*P* = 0.02], and median log_10_ CFU/mL change (−1.5 [IQR, −2.7 to −0.0] vs. 1.2 [IQR, 0.5 to 1.5], *P* = 0.03, respectively). Ampicillin 0.5 × MIC plus ceftriaxone demonstrated significantly more attainment of ≥2-log_10_ CFU/mL kill and median log_10_ CFU/mL change against penicillin-susceptible vs. borderline-PRASEF isolates (100% vs. 8.3% [*P* < 0.001], and median log_10_ CFU/mL change (−4.0 [IQR, −4.2 to −3.8] vs. −0.2 [IQR, −1.6 to 0.4], *P* < 0.001, respectively).

**Fig 3:**
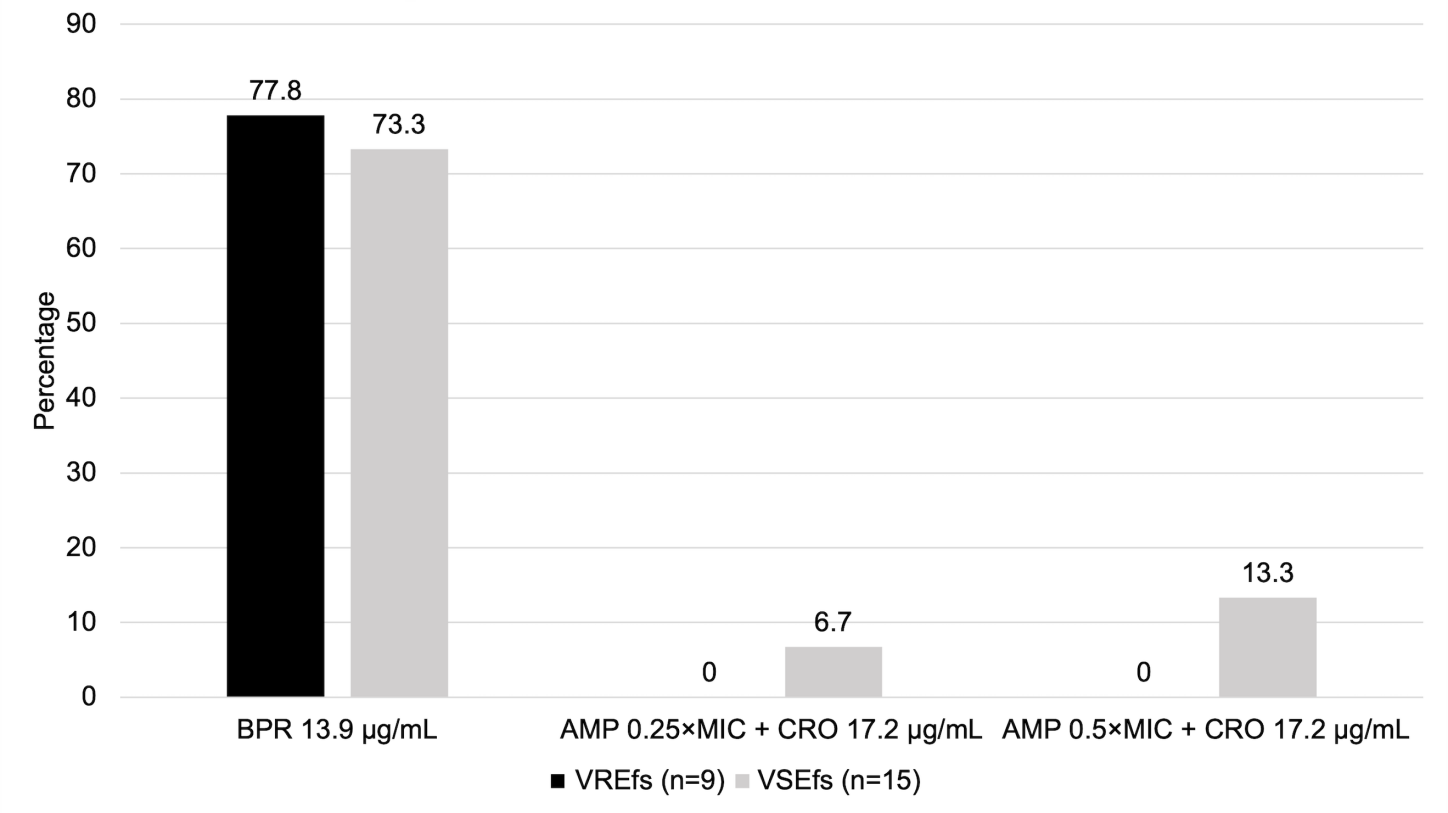
Percentage of vancomycin-resistant (VREfs) vs vancomycin-susceptible (VSEfs) borderline-PRASEF isolates in which ceftobiprole alone and ampicillin-ceftriaxone combinations achieved ≥2-log_10_ CFU/mL via 24-hour time-kill assays (*N* = 24). Abbreviations: AMP, ampicillin; BPR, ceftobiprole; CRO, ceftriaxone; VSEfs, vancomycin-susceptible *E. faecalis*. No significance was demonstrated between the activity of antimicrobials against VREfs vs VSEfs isolates.

Antimicrobial activity was compared among borderline-PRASEF isolates based on whether they were VREfs (*n* = 15) or VSEfs (*n* = 9) (Fig. 3). Ceftobiprole alone achieved similar ≥2-log_10_ CFU/mL kill against VSEfs vs. VREfs isolates (77.8% vs. 73.3%, *P* = 1.00, respectively) and median log_10_ CFU/mL change (−3.0 [IQR, −3.6 to −2.7] vs. −3.2 [IQR, −3.8 to −2.2]; *P* = 0.88, respectively). Ampicillin 0.25 × MIC and 0.5 × MIC plus ceftriaxone achieved similar ≥2-log_10_ CFU/mL kill against VSEfs vs. VREfs (0.0% vs. 6.7%, *P* = 1.00*;* 0.0% vs. 13.3%, *P* = 0.50, respectively) but were numerically much lower than ceftobiprole alone. The median log_10_ CFU/mL change for ampicillin 0.25xMIC plus ceftriaxone was worse in VREfs vs. VSEfs (1.5 [IQR, 0.7 to 1.7] vs. 0.6 [IQR, 0.3 to 1.2], *P* = 0.02) compared to ampicillin 0.5xMIC plus ceftriaxone which had similar activity (0.2 [IQR, −1.3 to 0.6] vs. −0.3 [IQR, −1.6 to 0.2], *P* = 0.34, respectively).

A post hoc analysis by ceftobiprole MIC compared differences in antimicrobial activity if the isolate ceftobiprole MIC exceeded tested concentrations (i.e*.,* 13.9 µg/mL; MIC ≤8 µg/mL [*n* = 22] vs. MIC ≥16 µg/mL [*n* = 8]). Isolates with a ceftobiprole MIC ≤8 µg/mL were more likely to achieve ≥2-log_10_ CFU/mL kill than isolates with a ceftobiprole MIC ≥16 µg/mL (90.9% vs. 50.0%, *P* = 0.03, respectively) as well as a greater median log_10_ CFU/mL change (−3.8 [IQR, −4.0 to −3.2] vs. −1.8 [IQR, −2.8 to −0.8], *P* < 0.001, respectively) (Table 1). Ampicillin-ceftriaxone had similar activity between both subsets of ceftobiprole MICs but was still lower than ceftobiprole alone.

**TABLE 1:** Activity of antimicrobials stratified by ceftobiprole MICs by those that exceeded the tested concentration (≥13.9 µg/mL) against *Enterococcus faecalis* isolates via 24-hour time-kill assays (*N* = 30)^*c*^

		Ceftobiprole MIC ≤8 µg/mL *n* = 22	Ceftobiprole MIC ≥16 µg/mL *n* = 8	*P*-value
≥2-log_10_ CFU/mL decrease, no. (%)	Ceftobiprole 13.9 µg/mL^*a*^	20 (90.9)	4 (50.0)	0.03
	Ampicillin 0.25 × MIC + ceftriaxone 17.2 µg/mL^*b*^	4 (18.2)	0	0.55
	Ampicillin 0.5 × MIC + ceftriaxone 17.2 µg/mL^*b*^	7 (31.8)	1 (12.5)	0.39
Median log_10_ CFU/mL change, [IQR]	Ceftobiprole 13.9 µg/mL^*a*^	−3.8 [−4.0, −3.2]	−2.0 [−2.8, −0.8]	<0.001
	Ampicillin 0.25 × MIC + ceftriaxone 17.2 µg/mL^*b*^	1.2 [−0.6, 1.5]	0.5 [0.2, 0.9]	0.64
	Ampicillin 0.5 × MIC + ceftriaxone 17.2 µg/mL^*b*^	−0.5 [−3.5, 0.2]	−1.3 [−1.8, −0.1]	0.91

^*a*^ Ceftobiprole was tested at the free plasma steady-state concentration (*f*Cp_SS_ = 13.9 µg/mL) based on population pharmacokinetic data for a 500 mg IV q8h regimen.

^*b*^ Ceftriaxone was tested at the free plasma steady-state concentration (*f*Cp_SS_ = 17.2 µg/mL) based on population pharmacokinetic data for a 2 g IV q12h regimen.

^*c*^ IQR, interquartile range; MIC, minimum inhibitory concentration.

**Fig 4:**
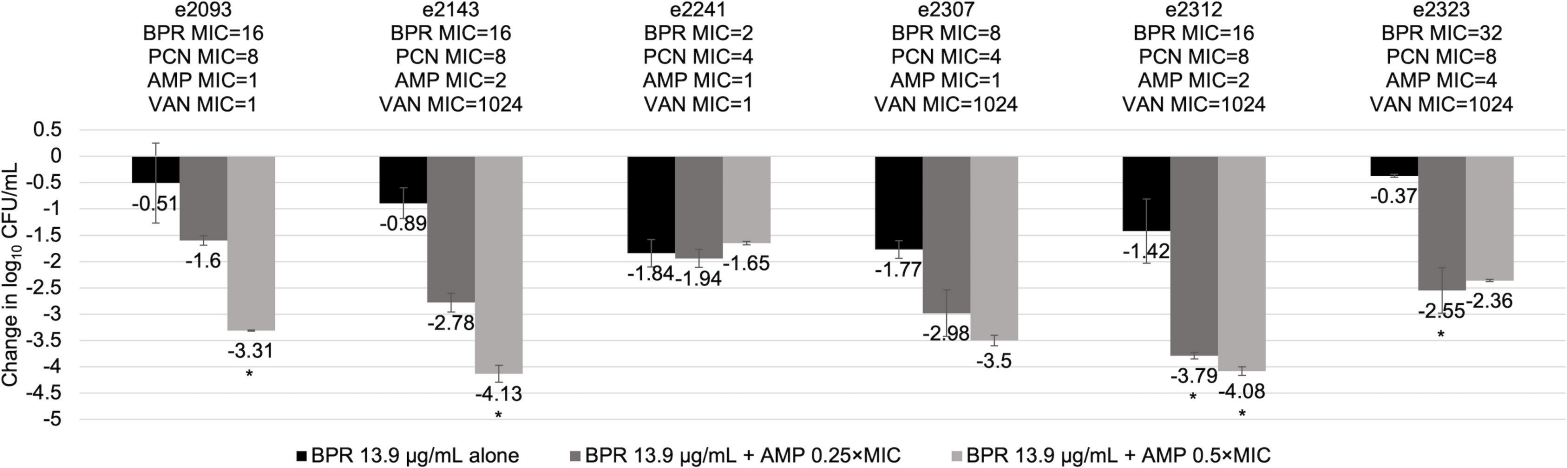
Activity of ampicillin-ceftobiprole combinations against six clinical *E. faecalis* blood isolates, which did not achieve ≥ 2-log_10_ CFU/mL kills with ceftobiprole alone via 24-hour time-kill assays. Abbreviations: AMP, ampicillin; BPR, ceftobiprole; MIC, minimum inhibitory concentration; PCN, penicillin; VAN, vancomycin. *Synergy (≥2-log_10_ CFU/mL decrease from the most active single agent) with ceftobiprole-ampicillin combination.

Ceftobiprole alone or ampicillin-ceftriaxone did not achieve ≥2-log_10_ CFU/mL kill in six isolates, all of which were borderline-PRASEF, and four had an elevated ceftobiprole MIC (MIC ≥16 µg/mL; Fig. 4). The six isolates were further tested against ampicillin 0.25 × MIC or 0.5 × MIC plus ceftobiprole, and ≥2-log_10_ CFU/mL kill was achieved in 66.7% and 83.3% of isolates, respectively. Synergy with ampicillin 0.5 × MIC plus ceftobiprole was seen in all four isolates with an elevated ceftobiprole MIC. It was observed that the higher ampicillin concentration (0.5 × MIC) had decreased activity compared to ampicillin 0.25 × MIC when combined with ceftobiprole against two isolates (e2241 and e2323) and therefore was further tested against ampicillin at 1 × MIC and 2 × MIC in combination with ceftobiprole (Table 2). A trend toward antagonism was observed in these two isolates.

**TABLE 2:** Activity of ampicillin-ceftobiprole combinations against *Enterococcus faecalis* isolates exhibiting growth at higher ampicillin concentrations^*b*^

	Antimicrobials	Isolate			
		e2241		e2323	
		Alone	Plus ceftobiprole	Alone	Plus ceftobiprole
Change in log_10_ CFU/mL from starting inoculum, mean ± SD	Ceftobiprole 13.9 µg/mL	−1.84 ± 0.26	–^*c*^	−0.37 ± 0.03	–^*c*^
	Ampicillin 0.25 × MIC	2.01 ± 0.03	−1.94 ± 0.17	0.30 ± 0.11	−2.55 ± 0.43^*a*^
	Ampicillin 0.5 × MIC	1.61 ± 0.34	−1.65 ± 0.03	−0.37 ± 0.31	−2.36 ± 0.02
	Ampicillin 1 × MIC	−1.62 ± 0.19	−0.89 ± 0.04	−3.47 ± 0.06	−2.42 ± 0.05
	Ampicillin 2 × MIC	−2.65 ± 0.23	−0.66 ± 0.01	−2.85 ± 0.31	−1.06 ± 0.13

^*a*^ Indicates a synergistic relationship between ceftobiprole and ampicillin (defined as ≥2-log_10_ CFU/mL decrease from the most active single agent).

^*b*^ MIC, minimum inhibitory concentration; SD, standard deviation.

^*c*^ “–” indicates not applicable.

## DISCUSSION

This study identified that ceftobiprole alone more frequently demonstrated *in vitro* activity against *E. faecalis* clinical blood isolates than ampicillin-ceftriaxone including against borderline-PRASEF and VREfs isolates. However, ceftobiprole alone did not achieve ≥2-log_10_ CFU/mL kill against six isolates, but the addition of ampicillin enabled activity in four of the six isolates.

Prior research demonstrated ceftobiprole maintains high susceptibility against *E. faecalis,* with MIC_50_s and MIC_90_s reported to range from 0.25 to 0.5 µg/mL and 1 to 4 µg/mL, respectively (20, 21, 23–27). Conversely, among our isolates, the MIC_50_ and MIC_90_ were higher, at 2 and 8 µg/mL, respectively, which may be attributed to our predominant inclusion of borderline-PRASEF isolates. One prior study of seven *E. faecalis* isolates with higher penicillin MICs observed higher ceftobiprole MICs in the five PRASEF compared to MICs in the two borderline-PRASEF isolates (range, 2–16 vs. 0.25-2 µg/mL, respectively) (14). While PRASEF isolates were not included in our study, we observed higher ceftobiprole MICs in our borderline-PRASEF cohort, which may be due to our larger sample size. While no breakpoint exists, ceftobiprole MICs ≥ 4 µg/mL have been described as non-susceptible in one study (14). Ceftobiprole non-susceptible strains are reported in less than 13% of isolates in Europe, the United States, Turkey, and Israel (23, 26, 27), whereas we report this in 44.3% of our isolates. Our cohort’s higher ceftobiprole MICs may be due to our smaller sample size and predominant inclusion of borderline-PRASEF isolates. The borderline-PRASEF phenotype is reported in up to 25% of isolates in New York, NY; however, determination of the true prevalence of borderline-PRASEF is still a challenge as penicillin MICs are not commonly reported against *E. faecalis* (17).

Comparing the relationship of ceftobiprole MICs to vancomycin MICs, we identified that the MIC_50_ and MIC_90_ were higher in VREfs isolates compared to VSEfs isolates (MIC_50_, 8 µg/mL vs. 1 µg/mL, respectively; MIC_90_, 16 µg/mL vs. 2 µg/mL, respectively). This contrasts with another study that found vancomycin resistance did not influence ceftobiprole susceptibilities (20). The authors tested 93 *E. faecalis* isolates from different countries, 17 of which were VREfs, and identified 94% of the VREfs isolates had a ceftobiprole MIC ≤1 µg/mL, compared to our results, which identified that 10% of VREfs isolates had a ceftobiprole MIC ≤1 µg/mL. This study did not report the penicillin MICs, so it is possible that a trend was seen in our data, as all of our VREfs isolates were borderline-PRASEF. Given the lack of studies, it remains unclear whether ceftobiprole MICs correlate with vancomycin resistance.

Ceftobiprole activity alone has been tested only in *in vitro* studies against *E. faecalis* (14, 20, 28). One study assessed ceftobiprole activity alone at 1 × MIC, 2 × MIC, and 4 × MIC via time-kill assays against seven *E. faecalis* isolates (five PRASEF, two borderline-PRASEF) (14). In five isolates (three, PRASEF; two borderline-PRASEF), ≥2-log_10_ CFU/mL kill was observed at ceftobiprole concentrations ≤ 16 mg/L. The remaining two PRASEF isolates only achieved ≥2-log_10_ CFU/mL kill when tested at ceftobiprole concentrations ≥32 mg/L. Ceftobiprole concentrations ≥32 mg/L are not physiologically achievable and may indicate the need for combination therapy (14, 29). The data support our findings that ceftobiprole alone can maintain activity against borderline-PRASEF but may require combination therapy with ampicillin in the setting of elevated ceftobiprole MICs.

Ceftobiprole activity has been evaluated in combination with different antimicrobials against *E. faecalis,* primarily in *in vitro* studies. Ceftobiprole has been demonstrated to have activity in combination with non-beta-lactam agents (i.e*.,* daptomycin, gentamicin) in small *in vitro* studies against VREfs isolates (20, 28). Ceftobiprole plus amoxicillin also demonstrated synergy in 10 out of 12 French *E. faecalis* IE isolates (no penicillin MICs included) in *in vitro* time-kills versus amoxicillin-ceftriaxone combinations, which achieved synergy in all 12 isolates (30). Conversely, we demonstrated that ampicillin-ceftobiprole had greater synergy than ampicillin-ceftriaxone, which may be due to our primary inclusion of borderline-PRASEF isolates. Ampicillin-ceftobiprole was also assessed in a retrospective, clinical study of 21 patients with invasive *E. faecalis* infections, 13 of whom had IE (21). Only patients with IE experienced an outcome, including death (*n* = 3), lack of microbiological eradication (*n* = 3), and relapse (*n* = 1). This study had an *in vitro* portion which demonstrated the patients’ isolates had low ceftobiprole MICs (MIC_50_, 0.5 µg/mL; MIC_90_, 1 µg/mL) with up to 80% of concentrations achieving 100%T > MIC and 60% achieving 100%T > 4 × MIC (31). Time-kill studies were completed against 10 isolates at inhibitory ceftobiprole concentrations (1 × MIC, 2 × MIC, and 4 × MIC), in which data for seven isolates are available. At 4 × MIC, 71% of isolates achieved bactericidal (≥3 log_10_ decrease in bacterial count at 24 hours) activity, and the remaining two isolates were tested in combination with ampicillin, and synergy (≥2 log_10_CFU/mL decrease by the combination compared with the most active antimicrobial) was demonstrated. This is similar to our findings, in which ceftobiprole alone had good activity, but in isolates in which activity was not high, ampicillin was able to provide additional activity.

We identified a trend toward antagonism in two isolates when increasing ampicillin concentrations were combined with ceftobiprole. Both isolates were borderline-PRASEF, one of which had an elevated ceftobiprole MIC (32 µg/mL) and was VREfs, while the other had a ceftobiprole MIC of 2 µg/mL and was VSEfs. It is unknown why ampicillin-ceftobiprole behaved antagonistically in these two isolates, as they are phenotypically different. We sought to identify ampicillin antagonism with ceftobiprole in six phenotypically similar isolates (data not shown); however, we were unsuccessful. Our failure in identifying antagonisms in phenotypically similar isolates may imply that there is a genetic component, which is influencing antagonism. Antagonism in ceftobiprole combination therapy has not been described in the literature.

The improved activity of ceftobiprole over ampicillin-ceftriaxone may be due to ceftobiprole binding to essential PBP-4 and non-essential PBPs (14). It is hypothesized in PRASEF isolates that beta-lactam susceptibility decreases from a mutation in the *pbp4* promoter region which leads to *pbp4* hyperexpression (14, 32). One such study demonstrated the potential of ceftobiprole to maintain bactericidal activity, even in the presence of these mutations in five isolates (14). While this mutation has not yet been described in borderline-PRASEF isolates, the *in vitro* phenomenon of decreased activity of ampicillin-ceftriaxone is known, and this may be why we observed an increase in activity in ceftobiprole combinations compared to ampicillin-ceftriaxone combinations (18). Furthermore, PRASEF isolates, which are also VREfs, were observed to have greater upregulation of PBP4 compared to PRASEF-VSEfs isolates (14). While one PRASEF-VREfs isolate expressed the promoter region mutation, the other lacked this, indicating another mechanism may lead to PBP4 overexpression; therefore, more studies are warranted to determine the genetic mechanisms in PRASEF-VREfs isolates.

An inherent limitation of time-kill assays is their static nature and inability to replicate physiologic pharmacokinetics. To counteract this limitation, we tested antimicrobials at physiologically achievable concentrations (*f*Cp_SS_). However, given the low ampicillin MICs in our isolates, physiological concentrations would well exceed MICs (*f*Cp_SS_, 29.2 µg/mL [33]; MIC range, 0.25–4 µg/mL; MIC_50_, 1 µg/mL; MIC_90_, 2 µg/mL) which would result in complete eradication of the isolates. Therefore, physiologic concentrations would make it difficult to detect synergy; thus, subinhibitory concentrations were more appropriate. Ceftobiprole was tested at the *f*Cp_SS_, but eight of our included isolates had ceftobiprole MICs above the *f*Cp_SS_ (13.9 µg/mL; MIC ≥16 µg/mL). This likely contributed to the reason why half of these isolates did not achieve ≥2-log_10_ CFU/mL with ceftobiprole alone, which may be why the synergistic effects of ampicillin 0.5 × MIC were necessary to achieve ≥2-log_10_ CFU/mL. Breakpoints are not yet established for ceftobiprole against *E. faecalis*, and given our findings, isolates with an elevated ceftobiprole MIC may indicate the need for combination therapy.

Overall, ceftobiprole alone demonstrated more activity compared to ampicillin-ceftriaxone combinations against *E. faecalis,* especially against borderline-PRASEF and VREfs isolates. In isolates that did not have ≥2-log_10_ CFU/mL kill with ceftobiprole alone, the addition of ampicillin was able to improve activity, especially in isolates with higher ceftobiprole MICs. It is unclear about the potential for ceftobiprole-ampicillin to demonstrate antagonism, as we observed a trend toward antagonism in two isolates, and more studies are warranted to determine the cause of this trend.

## MATERIALS AND METHODS

### Bacterial isolates

A total of 78 *E. faecalis* clinical blood isolates were selected from across a New York City health system. Seventy-one isolates were selected to be borderline-PRASEF (penicillin MIC 4–8 µg/mL), while the remaining seven isolates were penicillin-susceptible (penicillin MIC ≤2 µg/mL). All isolates were stored at −80°C in tryptic soy broth plus glycerol (CryoCare, Stamford, TX) and were sub-cultured once on brain heart infusion agar (BHIA; BD Difco, Sparks, MD) for 18–24 hours at 35°C prior to each experiment.

### Antimicrobials and media

Antimicrobial active pharmaceutical ingredients used included ampicillin sodium (Sigma-Aldrich Inc., Saint Louis, MO), penicillin G potassium (Sigma-Aldrich Inc.), and ceftriaxone sodium (Sigma-Aldrich Inc.) stored at 4°C, and ceftobiprole (Basilea Pharmaceutica International, Ltd., Allschwil) stored at −20°C. Antimicrobial solutions were made fresh for each experiment. Ceftobiprole was prepared per CLSI guidance using 110 µL of a 10:1 mixture of dimethyl sulfoxide (DMSO) to glacial acetic acid per 1.5 grams of ceftobiprole, followed by 15 minutes of vortexing before adding to 1 mL of water (15). To ensure there was no antimicrobial activity of the solvent, a 10:1 mixture of DMSO and glacial acetic acid was tested alone against a standard inoculum of ATCC 29212, which was unable to inhibit growth, in duplicate. BMD and time-kill assays were performed using cation-adjusted (calcium, 25  µg/mL; magnesium, 12.5  µg/mL) Mueller-Hinton broth (MHB; BD Difco, Sparks, MD). Isolate sub-cultures and viable cell counts to verify inoculums were plated to BHIA.

### Susceptibility testing

MICs were determined, in duplicate, for all 78 isolates against penicillin, ampicillin, ceftriaxone, and ceftobiprole via BMD per CLSI standards (15). Quality control strains were utilized to confirm antimicrobial activity: ATCC 29212 *E. faecalis* for penicillin, ampicillin, and ceftobiprole, and ATCC 29213 *Staphylococcus aureus* for ceftriaxone. Plates were incubated at 35°C and read at 20 hours. Isolates were categorized as susceptible or resistant to ampicillin or penicillin based on CLSI breakpoints (susceptible, ≤8 µg/mL; resistant, ≥16 µg/mL) (15). Penicillin-susceptible isolates were further categorized as borderline-PRASEF (penicillin MIC 4–8 µg/mL) or penicillin-susceptible (penicillin MIC ≤2 µg/mL). CLSI currently does not publish MIC breakpoints for ceftriaxone and ceftobiprole against *E. faecalis*. MICs were repeated if any MICs resulted in a range, wells were skipped, or the quality control or inoculum was out of range.

### Time-kill assays

Twenty-four borderline-PRASEF isolates and six penicillin-susceptible isolates were selected for comparison to be tested via time-kill analysis. Of the 24 borderline-PRASEF isolates, 15 were selected as they were VREfs. Each isolate was tested, in duplicate, against ampicillin, ceftriaxone, and ceftobiprole alone as well as ampicillin plus ceftriaxone combination via 24-hour static time-kill assays. Ampicillin was tested at subinhibitory concentrations (0.25 × MIC and 0.5 × MIC) as previously described (18, 34). Ceftriaxone was tested at the *f*Cp_SS_ of 17.2 µg/mL based on population pharmacokinetics for a 2 g intravenous injection every 12-hour regimen (t_½_=7.2 hours, *f*C_max_ = 28.9 µg/mL), as subinhibitory concentrations are not physiologically achievable due to enterococcus intrinsic resistance as previously described (35, 36). Ceftobiprole was tested at the *f*Cp_SS_ of 13.9 µg/mL based on population pharmacokinetics for a 500 mg intravenous injection every 8-hour regimen (t_½_=3.1  hours, *f*C_max_ = 29.2  µg/mL 29), as ceftobiprole MICs are reported to be higher in isolates with elevated penicillin MICs and therefore subinhibitory concentrations may not be physiologically achievable (14). Twelve-well plates were utilized with a final volume of 2 mL in each well. Each *E. faecalis* isolate was prepared as a 0.5 McFarland for a target starting inoculum of 10^6^ ± 0.3 CFU/mL. Plates were incubated at 35°C and placed on an orbital shaker at 50 rotations per minute. Samples were taken at 0, 4, and 24 hours and subsequently diluted in normal saline and plated to BHIA. Samples were incubated for 18–24 hours at 35°C to obtain viable cell counts. Experiments were repeated if a standard deviation of ≥1-log_10_ CFU/mL resulted. The lower limit of detection was 2-log_10_ CFU/mL.

Our primary objective was to determine the difference in activity of ceftobiprole alone versus ampicillin-ceftriaxone combination in borderline-PRASEF isolates. A subanalysis was completed comparing VREfs vs VSEfs borderline-PRASEF isolates, as VREfs was only present in borderline-PRASEF isolates. We further completed a post hoc analysis based on ceftobiprole MICs and compared isolates with an MIC ≤8 µg/mL to those with an MIC ≥16 µg/mL, as this was the cut-off of the simulated ceftobiprole concentration, which was tested (13.9 µg/mL). We measured activity based on isolates with ≥2-log_10_ CFU/mL decrease from the initial inoculum at 24 hours. If ceftobiprole alone did not achieve ≥2-log_10_ CFU/mL decrease, the activity of ceftobiprole in combination with ampicillin was tested. Synergy was measured at 24 hours as a ≥ 2-log_10_ CFU/mL decrease from the most active single agent (37). Antagonism was measured at 24 hours as a ≥ 2-log_10_ CFU/mL increase from the most active single agent. Median log_10_ CFU/mL change after 24 hours from the initial starting inoculum was calculated for each isolate.

### Statistical analysis

All statistical analyses were completed using R Studio (R version 4.1.2 [http://www.R-project.org/]). Categorical variables were assessed by chi-square or Fisher’s exact test as appropriate, and continuous variables were assessed with the Wilcoxon Rank Sum. In both cases, statistical significance was determined by a two-sided *P*-value of <0.05.

## SUPPLEMENTAL MATERIAL

Table S1 aac.01050-25-s0001.tiff MICs of *E. faecalis* clinical blood isolates by broth microdilution.

ASM does not own the copyrights to Supplemental Material that may be linked to, or accessed through, an article. The authors have granted ASM a non-exclusive, world-wide license to publish the Supplemental Material files. Please contact the corresponding author directly for reuse.
