# Supplementary figures and images for: Ceftobiprole alone versus ampicillin-ceftriaxone against borderline-penicillin-resistant, ampicillin-susceptible, and vancomycin-resistant Enterococcus faecalis isolates
Source: Antimicrob Agents Chemother. 2025 Nov 24;70(1):e01050-25. doi: 10.1128/aac.01050-25 (PMC12777555; doi:10.1128/aac.01050-25)

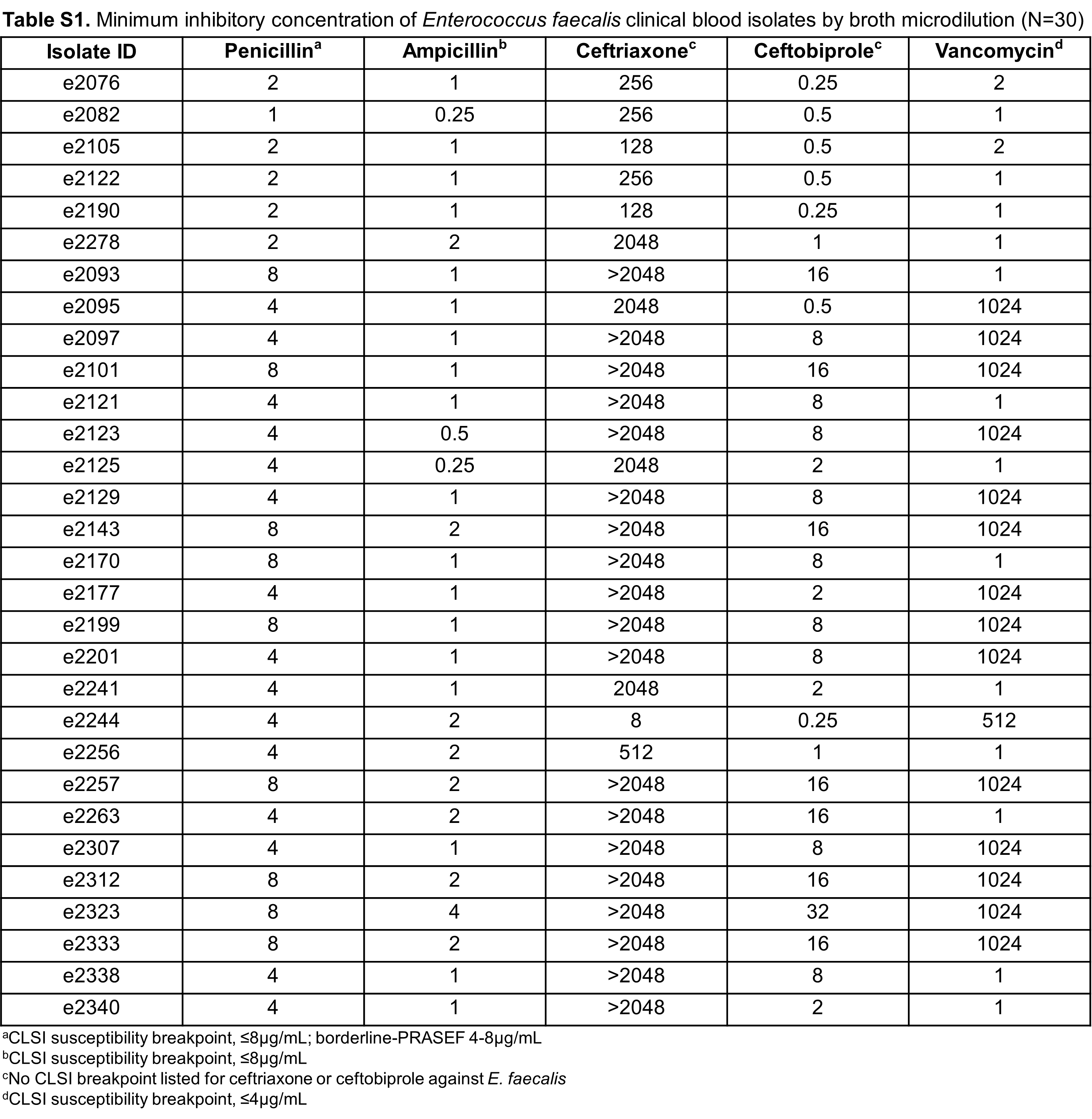

Supplement: Table S1 — MICs of E. faecalis clinical blood isolates by broth microdilution. [file aac.01050-25-s0001.tiff]
